# Sport-Specific Rehabilitation, but Not PRP Injections, Might Reduce the Re-Injury Rate of Muscle Injuries in Professional Soccer Players: A Retrospective Cohort Study

**DOI:** 10.3390/jfmk7040072

**Published:** 2022-09-21

**Authors:** Eduard Bezuglov, Vladimir Khaitin, Maria Shoshorina, Mikhail Butovskiy, Nikita Karlitskiy, Evgeny Mashkovskiy, Evgenii Goncharov, Bekzhan Pirmakhanov, Ryland Morgans, Artemii Lazarev

**Affiliations:** 1 Department of Sport Medicine and Medical Rehabilitation, First Moscow State Medical University Named after I.M. Sechenov (Sechenov University), 119435 Moscow, Russia; 2 High Performance Sports Laboratory, Moscow Witte University, 115432 Moscow, Russia; 3 Sirius University of Science and Technology, 354349 Sochi, Russia; 4 Department of Sports Medicine and Medical Rehabilitation, Pavlov First State Medical University, 197022 Saint-Petersburg, Russia; 5 Academy of Talents, 121552 Moscow, Russia; 6 Medical Faculty, Synergy University, 125190 Moscow, Russia; 7 Central Clinical Hospital of the Russian Academy of Sciences, 117593 Moscow, Russia; 8 Russian Medical Academy of Continuous Professional Education, 125993 Moscow, Russia; 9 Department of Epidemiology, Biostatistics and Evidence-Based Medicine, Faculty of Medicine and Health Care, Al-Farabi Kazakh National University, Almaty 050040, Kazakhstan; 10 Football Club Kairat, Almaty 050054, Kazakhstan; 11 Department of Internal Medicine, Mount Sinai Hospital, Chicago, IL 60608, USA

**Keywords:** PRP, muscle injury, specific rehabilitation, football (soccer)

## Abstract

Platelet-rich plasma (PRP) injections are extremely popular in the management of sports injuries in elite athletes. However, data on the use of various administration protocols of PRP are contradictory. The efficacy of platelet-rich plasma in the treatment of muscle injuries in professional soccer players has to be contextualized within the sport-specific rehabilitation program. Despite the questionable role of PRP, a well-structured rehabilitation program is still regarded as the gold standard. We examined the efficacy of various PRP protocols in the management of muscle injuries in professional soccer players in respect to treatment duration and injury recurrence. A retrospective cohort study. Muscle injuries in professional soccer players (*n* = 79, height 182.1 ± 5.9 cm, weight 76.8 ± 5.8 kg, BMI 23.1 ± 1.4 kg/m^2^) from three elite soccer clubs from the Russian Premier League were recorded during the 2018–2019 season. The injuries were graded based on MRI, using the British Athletic Muscle Injury Classification. Treatment protocols included the POLICE regimen, short courses of NSAID administration, and the specific rehabilitation program. The sample group of players were administered PRP injections. The average treatment duration with PRP injection was significantly longer than conventional treatment without PRP, 21.5 ± 15.7 days and 15.3 ± 11.1 days, respectively (*p* = 0.003). Soccer-specific rehabilitation and obtaining MRI/US before the treatment was associated with significantly reduced injury recurrence rate (*p* < 0.001). There was no significant difference between the PRP injection protocol applied to any muscle and the treatment duration in respect of grade 2A–2B muscle injuries. The total duration of treatment of type 2A–2B injuries was 15 days among all players. In the group receiving local injections of PRP, the total duration of treatment was 18 days; in the group without PRP injections, the treatment duration was 14 days. In our study, PRP treatment was associated with longer treatment duration, regardless of which muscle was injured. This may reflect the tendency to use PRP in higher-degree injuries. Soccer-specific rehabilitation significantly reduced the injury recurrence rate when compared to the administration of PRP injections. MRI/US imaging before returning to play was also associated with a lower injury recurrence rate. There was no significant difference between the PRP injection protocol applied to any muscle and the treatment duration in treatment of type 2A–2B muscle injuries.

## 1. Introduction

Muscle injuries account for 20–46% of all injuries in professional soccer [1,2]. A squad of 25 players can expect 15 muscle injuries per season [1,3]. These are mostly non-contact injuries, and the hamstring muscles are often most affected, followed by the adductor muscle group, quadriceps, and calf muscles [4]. Hamstring injury results in a median recovery period of 14 days, or 3–5 missed matches per season, with the average recurrence rate is 16–18% [1,5].

Ultrasonography and magnetic resonance imaging (MRI) imaging [6,7] are commonly used for diagnostic purposes. Ultrasonography is widely used due to its convenience, and suitability for follow-up examinations, as well as ultrasound-guided injections [8]. However, its prognostic value is limited by examiner-dependent factors [9]. Thus, MRI imaging is considered the ‘gold standard’ as it can be used for clear classification of the injury, confirming its location and describing the extent of any underlying structural damage (e.g., muscle fiber disruption, edema, hematoma) [10], and therefore, may help predicting the duration of treatment of muscle injuries [11], as well as affect the return to play decisions [12].

Modern muscle injury classification systems [13,14] comprise of clinical and imaging data, which enables the evaluation of injury severity and treatment duration prognosis. This allows a rehabilitation plan to be designed and administered in regards to the healing time of the damaged tissue. The widely recognized RICE protocol of rest, ice, compression, and elevation is recommended for early management, while the POLICE (protection, optimal loading, ice, compression, and elevation) paradigm [15] highlights the need for safe and effective loading in acute soft tissue injury management. Additionally, a significant component of the rehabilitation process is a timely introduction to a series of sport-specific exercises [16]. While nonsteroid anti-inflammatory drugs (NSAIDs) can exert negative effects on muscle regeneration [17], they are widely used in the management of muscle injuries [18].

Several studies demonstrated the clinical efficacy of local platelet-rich plasma (PRP) administration for muscle injury treatment [19,20,21,22,23]. The effect of PRP is mediated by the platelet alpha-granules, which contain various growth factors, e.g., FGF-2, HGF, and TGF-β1. These growth factors stimulate tissue repair, and potentially mitigate pain, edema, and shorten overall treatment duration [24,25,26]. Nonetheless, contradictory findings regarding the advantages of PRP over conventional therapies have been reported [24,27]. A major limitation of this method is that no consistent methodology for muscle injury treatment has been described, and notably, the concentration of platelets in PRP products varies broadly and might be not reproducible even in the same individual using the same PRP preparation kit [28].

Therefore, the development of muscle injury treatment protocols is of great practical interest in professional soccer. In that sense, more data are needed on various aspects of this treatment such as effects of PRP, sports-specific rehabilitation etc. on treatment duration and recurrence rate. We hypothesized that there would be no significant difference in the return to play time and injury recurrence rate in elite professional soccer players who had sustained muscle injury and that either received PRP injection or traditional conservative management. Separately, we performed analysis of the treatment of grade 2A-2B muscle injuries.

## 2. Methods

### 2.1. Participants and Study Design

A retrospective analysis of muscle injuries was conducted using the data from three elite soccer clubs from the Russian Premier League (RPL) during the 2018–2019 season. Seventy-nine professional soccer players (mean age 24 ± 7 years, height 182.1 ± 5.9 cm, weight 76.8 ± 5.8 kg, BMI 23.1 ± 1.4 kg/m^2^) formed the sample. The players from these soccer teams were members of their respective national teams and regularly participated in the RPL and other UEFA soccer tournaments.

The inclusion criteria for this study were as follows:

- Age of 18 years and older,
- Signed informed consent,
- Time period from injury event >3 days.

The treatment duration was defined as the time period between the injury event and return to play. If a player returned to play after a short period (1–3 days) of rest and treatment, his case was not included in the analysis. Treatment outcomes of lower leg muscle injuries were examined. Ethical approval for the study was granted by the Ethics Committee of Sechenov University (N 08-19 dated 05.06.2019).

### 2.2. Classification of Muscle Injuries

The injuries were graded based on MRI reporting, using the British Athletic Muscle Injury Classification [14]. The 1.5 T MRI scans were performed utilizing T2-weighted fat-suppressed spin echo sequences. The injury severity was assessed independently by two radiologists with at least 10 years of experience working with musculoskeletal MRI. All images were processed with eFilm Workstation (Version 4.2.2; IBM, Armonk, NY, USA), and saved for later analysis. All diagnostic tests were completed within 24–48 h from the injury event. An injury in the same anatomical region as the previous injury was considered a recurrence if it occurred within 2 months of the last day of rehabilitation after the primary injury. We used even more stringent criteria, and deliberately extended this period to 6 months. Therefore, we considered a recurrent muscle injury in the muscle group originally affected, which resulted in missing at least one training day within 6 months of the original injury.

### 2.3. Treatment Protocols

Treatment protocols included the POLICE regimen, short courses of NSAID administration, and the soccer-specific rehabilitation program. The POLICE treatment regimen started immediately after the injury event, and continued for the following 3 days. It included intermittent pneumatic compression cryotherapy, applied 7–8 times per day, lasting 15–20 min (Game Ready^®^ CoolSystems Inc., Concord, CA, USA). Compression cryotherapy was also applied after each rehabilitation session. NSAIDs were administered for 3–5 days, namely Ibuprofen 400 mg two times per day and Etoricoxib (ARCOXIA^®^) Merck & Co., Kenilworth, NJ, USA, 90 mg two times per day after a meal.

### 2.4. PRP Applications

The decision to apply PRP in treatment was made independently by the team physician of each of the three soccer teams. The type of injury according to the British Athletic Muscle Injury Classification, injury location, and other individual player- and sport-specific factors were considered.

PRP was obtained by centrifuging the blood utilizing the Endoret^®^ (PRGF^®^) Centrifuge System IV (BTI Biotechnology Institute, San Antonio, Spain) at 1902 rpm for 8 min. The platelet count was 600–700,000/mL, White Blood Count (WBC) was minimal (leucocyte-poor PRP). The quantity of calcium chloride to activate the platelets was 50 μg.

Three different PRP-injection protocols were used in 34 players. The protocols were as follows: (i) single PRP injection of 8–10 mL (*n* = 12); (ii) three PRP injections of 3–5 mL (*n* = 6) with an interval of 5–7 days between injections; (iii) three PRP injections of 8–10 mL (*n* = 16) with an interval of 5–7 days between injections.

### 2.5. Rehabilitation Program

The rehabilitation training was initiated the day following diagnostic imaging examinations and the first application of PRP. In every case, training sessions were performed by the specially trained physiotherapist. The training session was conducted daily and lasted approximately 100 min. It included cycling exercises, resistance band, and leg swinging exercises, which simulated the biomechanics of soccer actions. The final phase of rehabilitation was performed in a soccer-specific environment on a natural field and monitored using a Global Positioning Satellite (GPS) tracking system (WIMU PRO). The WIMU PRO device (Realtrack Systems, Almería, Spain) is comprised of different sensors, including four accelerometers, three gyroscopes, a magnetometer, a global navigation satellite system chip (GNSS; M = 8.96; SD = 1.56) and a UWB chip [29]. Specifically designed vests were used to hold the devices, located on the player’s upper torso, and anatomically adjusted to each player, as previously described. The ability to perform sprinting, which is equivalent to 20 min of a conventional soccer match, was a pre-requisite to allow the player to return to play.

### 2.6. Statistical Analysis

The database was created with Microsoft Excel software; statistical analysis was performed utilizing the IBM SPSS 23.0 (Armonk, NY, USA). Continuous data were tested for normality of distribution with the Kolmogorov–Smirnov test. Normally distributed data were described with mean (M) and standard deviation (SD). Median (Me) and quartiles were used in case of abnormal distribution). Percentage and absolute numbers were provided for categorical data. Mann–Whitney U test was performed to compare the duration of treatment and recovery before return to play between athletes with and without pulmonary lesions. Spearman’s correlation was used for non-normal distributed data. Results were considered statistically significant at *p* < 0.05.

## 3. Results

The average duration of injury treatment for all locations and all grades was 18.8 ± 14.1 days. The average treatment duration with PRP administration was 21.5 ± 15.7 days, and 15.3 ± 11.1 days without PRP (*p* < 0.05). The injury recurrence rate was 10.1%. When analyzing specific muscle groups, adductor muscle injuries comprised 65.8% of all muscle injuries, hamstring, calf, and quadriceps muscle injuries comprised 19%, 11.4%, and 3.8% respectively. The treatment duration for the adductor, hamstring, calf, and quadriceps muscle injuries was 18.3 ± 14.1, 16.2 ± 8.3, 15.7 ± 12.5, and 27.1 ± 20.4 days, respectively. Type 2A–2B muscle injuries accounted for 84.8% of all (67 of 79) injuries reviewed.

### 3.1. Analysis of Factors Influencing Treatment Duration

As the treatment duration was not normally distributed, non-parametric analysis with Kruskal–Wallis and Mann–Whitney tests were performed to compare treatment duration in players imaging before returning to play. Overall, the use of PRP was associated with longer treatment duration. Players with longer treatment duration underwent MRI or MRI + US imaging more frequently compared to players with shorter treatment durations, who underwent only US or no imaging (Table 1). There was no correlation between injury location and treatment duration. Sport-specific rehabilitation was not associated with shortened duration of the treatment.

### 3.2. Analysis of Factors, Influencing Injury Recurrence Rate

Soccer-specific rehabilitation significantly decreased injury recurrence rate. Players who underwent MRI/US imaging before returning to play also demonstrated a lower injury recurrence rate (Table 2). Other factors, including PRP-injections did not seem to affect re-injury rate.

### 3.3. The Effect of Different PRP Application Protocols in the Treatment of 2A–2B Muscle Injuries

In total, 67 players had type 2A–2B injuries; 34 players received PRP injections, while 33 underwent conventional treatment. Twelve people received PRP once in a volume of 8–10 mL, and six people received one injection every 5–7 days (a total of 2–3 injections of PRP in a volume of 3–5 mL). Sixteen people received one injection every 5–7 days (a total of 2–3 injections of PRP in a volume of 8–10 mL). The total duration of treatment was 15 days among all players. In the group using local injections of PRP, the total duration of treatment was 18 days; in the group with no use of PRP the treatment duration was 14 days. Recurrences were seen in 10% of cases among all players. In the group using PRP, recurrences were seen in 9% and in the group without using PRP in 12%.

The Mann-Whitney test was performed to compare treatment duration in groups that received different PRP injection protocols. A longer treatment duration was reported in players who received three PRP injections of 8–10 mL with an interval of 5–7 days between injections in comparison to players who did not receive PRP injections. A significantly lower injury recurrence rate was observed in players that were administered three injections of 8–10 mL of PRP with an interval of 5–7 days between injections comparing to those who received a single PRP injection of 8–10 mL (chi-square test, *p* = 0.021). No significant differences were observed between players that received three PRP injections of 8–10 mL with an interval of 5–7 days between injections and players who did not receive PRP injections (*p* = 0.09). There was no significant difference between the PRP injection protocol applied to any muscle and the treatment duration (Kruskal–Wallis test, *p* > 0.05).

## 4. Discussion

The most relevant clinical finding of the present study is that soccer-specific rehabilitation significantly lowered the re-injury rate when compared to the administration of PRP injections. PRP injections were associated with longer duration of treatment, however it may reflect the tendency to use PRP in higher-degree injuries. In the present investigation, the treatment duration and the recurrence rate of muscle injury was lower than previously reported in similar studies [1,5].

About 16% of muscle injuries in professional football are recurrent injuries [1]. In leading European soccer clubs, the average treatment duration for lower limb muscle injuries is 14 days, with an injury recurrence rate of 16% [1,5]. Treatment duration in hamstring injuries is 28 days in 14% of soccer players, while treatment duration of re-injuries is 30% longer than the treatment of primary injuries. Re-injuries mostly affect the biceps femoris muscle (18%), while in the semimembranosus and semitendinosus muscles re-injuries occur only in 2% of soccer players [1,5].

Previous studies found that the return to play was influenced by several factors, including injury mechanism, severity, and imaging findings [30]. Whether or not PRP shortens the return to play period is questionable. A double-blind randomized placebo-controlled study showed no effects of PRP injections on the return to play of Qatari athletes with grade 1–2 hamstring injuries, with a treatment duration of 21–27 days regardless of the use of PRP injections, with a recurrence rate of 8–11% [27]. Additionally, a recent meta-analysis that included only randomized placebo-controlled studies showed no advantages of PRP injections in the management of muscle injuries [31]. However, it should be noted that different PRP injection protocols were used in included studies and the participants from several studies were not professional soccer players.

The grade of injury diagnosed with MRI scanning does have a prognostic value [4], while modern classification systems provide clinically relevant information to plan treatment duration [32]. The changes on MRI at the time of injury correlate with the time to return to regular to sport in grade 1 and 2 injuries, but MRI alone cannot be used as a criterion for returning to regular training and minimizing the risk of re-injury. Even in the absence of any changes on MRI, the rate of recurrence may reach 27% [12]. On the other hand, persistent MRI changes (an intramuscular increase in the signal intensity) are present in up to 89% of athletes who had successfully returned to sport with no clinical symptoms. Hence, normalization of MRI appearance is not required for a safe return to regular training [33], and intramuscular fibrosis can observe in almost one third of athletes with no association with recurrence.

The criteria used for return to play after injuries of the lower limb muscles vary, and include “achievement of a pre-traumatic level of activity”, “the ability to fully engage in sports”, “no pain”, “similar strength”, “similar flexibility”, “clearance by medical personnel”, “functional efficiency”, “reaching a pre-traumatic level of activity”, and “being able to fully engage in sports” [34]. Using MRI appearance as a criterion for safe return to play does not eliminate the risk for injury recurrence [32], and other modalities, such as isokinetic testing or ultrasonography, do not exclude the risk of recurrence even when normality has been restored [16]. In this respect, it is not surprising that the ratio between eccentric strength of hamstring muscles and concentric strength of the quadriceps muscle can only be considered as weak risk factors for hamstring muscle injuries [35]. Equally, there is only a weak association between the risk of lower limb muscle injuries and isokinetic testing performed before the competitive season by footballers of 14 professional teams [36].

Our study has a number of limitations: the fact that three different PRP injections protocols were used in our study is one of them. The choice of the regimen used was chosen by the sports medicine physician in each of the three teams involved in the present investigation, and remained constant during the study period. Second limitation includes the lack of clear criteria for PRP application in each case. It is worth mentioning that such decisions are usually made by team physicians individually after considerations of multiple player- and roster-specific factors and it is hard to apply strict criteria.

Future studies need to assess the various protocols and indications of sports medicine physician for PRP use with clearer inclusion criteria for PRP use to better understand the patterns of its application. That would clarify whether PRP is more frequently used in higher-degree injuries. The detailed study of the effects of sport-specific rehabilitation in regards of treatment duration and recurrence rate are warranted.

## 5. Conclusions

In our study, PRP treatment was associated with longer treatment duration, regardless of which muscle was injured. It may reflect the tendency to use PRP in higher-degree injuries. Soccer-specific rehabilitation significantly reduced the injury recurrence rate when compared to the administration of PRP injections. MRI/US imaging before returning to play also was associated with a lower injury recurrence rate. There was no significant difference between the PRP injection protocol applied to any muscle and the treatment duration in treatment of type 2A–2B muscle injuries. 

## Figures and Tables

**Table 1:** The association between various factors and the duration of treatment (Mann–Whitney test for two categories; Kruskal–Wallace test for several categories).

Factor	*p*-Value
PRP injections	0.003
MRI or US imaging obtained	<0.001
NSAID use	0.665
Degree of injury	<0.001
Sport-specific rehabilitation	0.321

**Table 2:** The effect of treatment methods, injury severity, and imaging modality on injury recurrence rate (Pearson’s chi-squared test).

Factor	*p*-Value
PRP injections	0.675
MRI or US imaging obtained	0.025
NSAID use	0.201
Degree of injury	0.445
Sport-specific rehabilitation	0.001

## Data Availability

The data presented in this study are available on request from the corresponding author.
